# Failure of preventive treatments in migraine: an observational retrospective study in a tertiary headache center

**DOI:** 10.1186/s12883-020-01839-5

**Published:** 2020-06-30

**Authors:** Marianna Delussi, Eleonora Vecchio, Giuseppe Libro, Silvia Quitadamo, Marina de Tommaso

**Affiliations:** Applied Neurophysiology and Pain Unit, Basic Medical Sciences, Neuroscience and Sensory System Department-SMBNOS, Bari Aldo Moro University, Policlinico General Hospital, Giovanni XXIII Building, Via Amendola 207, A 70124 Bari, Italy

**Keywords:** Migraine, Preventive treatment, Observational study, Tertiary headache center

## Abstract

**Background:**

Although the criteria for acute migraine treatment and prevention have been well described, there are still unmet needs, general underuse and low benefits of preventive drugs.

The aim of the present study was to retrospectively observe the short-term effect of preventive treatment in a cohort of migraine patients attending a tertiary headache center, using data from electronic medical records.

**Methods:**

This was an observational retrospective cohort study based on data collected in a tertiary headache center. Data were extracted from an electronic dataset collected from January 2009 to December 2019. The main selection criteria were as follows: age of 18–75 years; diagnosis of migraine without aura (MO), migraine with aura (MA) or chronic migraine (CM); a control visit 3 months after the first access; and prescription of preventive treatment with level of evidence 1 as reported by Italian guidelines. As the primary outcome, we considered the change in the frequency of headache at the follow-up visit. Then, as secondary outcome measures, we used disability scores, intensity of headache, and allodynia. As predictive factors, we considered age, migraine duration, sex, headache frequency, allodynia, anxiety and depression at baseline, and comorbidity with fibromyalgia.

**Results:**

Among the 6430 patients screened, 2800 met the selection criteria, 1800 returned to the follow-up visit, 550 withdrew because of adverse events, and 1100 were included the analysis. One hundred thirty-four patients had a frequency reduction of 50% or more. Flunarizine was used for less severe migraine, with a better effect compared to those of other drugs (odds ratio: 1.48; p: 0.022). Low headache frequency and absent or mild allodynia predicted a better outcome.

**Conclusions:**

The mild effect of preventive drugs on migraine features and even the number of patients who were lost to follow-up or dropped out because of adverse events confirm that in severe and chronic patients, the first line of prevention can only delay a more focused therapeutic approach.

## Background

Migraine is a common and disabling disease, affecting approximately 12–14% of the occidental population [1]. Despite the criteria of acute episodes, treatment and prevention having been well described [2], there is still a large proportion of patients with unmet needs, general underuse and low benefits of preventive treatments [3]. National guidelines indicate the conditions for the prescription of preventive treatment, the drugs with evidence of action and consequent recommendations for their use [2]. Real-world studies have reported the modality of preventive treatment prescription for general practitioners and neurologists. An observational study in the U.S. included 43,660 migraine patients receiving different preventive drugs in addition to acute treatment [4]. The study identified the main comorbidities associated with preventive drug prescription, such as sleep disorders in females, but there are no data on specific treatments and their effects. The French SMILE study assessed the determinants of the prescription of migraine preventive therapy by GPs and neurologists and factors determining eligibility, such as the frequency and severity of headache and scarce evidence of the efficacy of acute treatment [5]. An Italian study evaluating the use of triptans showed that only 21.3% of patients using triptans were under oral preventive treatment or botulin toxin treatment. In the same population, amitriptyline was the most prescribed drug, followed by topiramate (6.3%), propranolol (3.3%) and atenolol (2.7%). The rate of improvement was estimated on the basis of the reduction in triptan use; the use of triptans was even significantly lower among subjects treated with oral preventive therapies than among those without these drugs, though mild improvement was present in the group with chronic migraine [3]. The study underlined that the current use of preventive therapies is limited and has negligible benefits and that most migraine patients currently have unmet needs.

Few data are available about preventive treatment prescriptions and their efficacy in patients observed at third-level headache centers. Such data could potentially be useful for understanding the utility of available preventive drugs and the real need for new drugs for migraine prevention [6].

The aim of the present study was to retrospectively observe the short-term effect of preventive treatment in a cohort of migraine patients attending a tertiary headache center, using data from electronic medical records.

## Methods

### Study design

This was an observational retrospective cohort study based on data collected in a tertiary headache center.

### Setting

The Applied Neurophysiology and Pain Unit (ANPlab) includes three neurologists, two psychologists and one nurse. All patients are asked to keep a headache diary at the time of booking the first visit (which precedes the first access by approximately 3 months) and to return 3 months later for a follow-up visit, regardless of the prescription of preventive treatment.

An example of a headache diary used by patients is reported in Supplementary Fig. 1. The diary includes the allodynia scale with scores from 0 to 12, according to previous studies [7].

Only a limited number of clinical features are converted into electronic codes useful for retrospective analysis.

The local Ethics Committee of Bari Policlinico General Hospital approved the use of the electronic database, and patients signed an informed consent form about the inclusion of their data and use for scientific purposes.

### Participants

The present data were extracted from an electronic dataset collected from January 2009 to December 2019.

For the present analysis, we selected patients aged 18–75 years; who received a diagnosis of migraine without aura (MO), migraine with aura (MA) or chronic migraine (CM) [8, 9]; who were currently free from preventive treatments and the use of central nervous system-targeting drugs; who had had a control visit 3 months after the first access; and who were prescribed preventive treatment with level of evidence 1 as reported by Italian guidelines [2]. We did not select patients with severe general medical comorbidities, such as hepatic, renal and cardiovascular insufficiency; previous or current neurologic diseases beside migraine; or a diagnosis of current or previous psychiatric diseases. *Exposure* All the preventive drugs with level of evidence 1, including antidepressants (amitriptyline), beta-blockers (propranolol and atenolol), calcium channel blockers (flunarizine), and antiepileptic drugs (topiramate), were initially considered together and later analyzed individually in subgroups treated with the respective drugs. Meanwhile, data analysis was performed, and we noted that for a subgroup of patients who specifically denied the use of drugs in a first attempt, magnesium was prescribed. In another subgroup, clinicians opted for the use of candesartan because of the presence of hypertension and contraindication to the use of beta-blockers. In the final analysis, we thus decided to also include the patients described above, although neither treatment was included in the list of drugs with level of evidence I. Considering that valproate is not indicated in Italy for the treatment of migraine, clinicians prescribed topiramate as the first preventive drug. Other drugs with level of evidence II, such as gabapentin, pizotifen, and dihydroergotamine, were not used as primary preventive treatments [2].

### Variables

As a primary outcome measure for the effect of preventive treatment, we considered the change in the frequency of headache at follow-up. As secondary outcomes, we used the MIDAS score [10], the intensity of headache on a 0–10 scale, allodynia [7] and general quality of life [11]. For headache frequency, we considered the average number of headache days per month over the last 3 months. As predictive factors for headache frequency reduction, we considered age, gender, duration of headache, frequency and intensity of headache, allodynia, and anxiety and depression scores as observed at the first access [12, 13]. Comorbidity with fibromyalgia was also considered, as the center has specific experience with this condition [14]. The lack of distinction between headache and migraine days could be potentially confusing for the evaluation of the outcome; this feature was introduced as an option in the electronic database over the course of clinical activity. Another confusing factor could be FM comorbidity, which could have influenced the choice of preventive treatment. A source of potential bias is the unpredictable number of patients lost to follow-up, the low reliability of headache diaries, and the choice of treatments other than those recommended as first-line drugs, which may happen in clinical practice. Another bias could be the lack of data about the number of migraine days and the number of analgesics used. Data on symptomatic drugs and their effects are missing because of a problem in the electronic database occurring from 2009 to 2016.

Patients with missing data for the variables considered primary and secondary outcome measures were not considered in the present analysis.

## Statistical analysis

Considering that an average reduction in migraine frequency of approximately 50% is generally indicated as a good outcome and that a reduction lower than 30% could be interpreted as treatment failure, we assessed a minimum sample size of 459 patients to explore the general effect of preventive treatments (β of 0.20–80%, power of α i 0.05). This was valid for the entire migraine group.

Preliminarily, univariate ANOVA was performed for the primary variable, comparing the percent change in headache frequency among single migraine types and drug subgroups. The number of cases with a 50% change in migraine frequency was computed for the whole group and in each subgroup treated with a specific drug, thus computing the odds ratio to establish the superiority of a drug within the whole migraine group. Multivariate ANOVA was also performed to assess the changes in secondary outcome variables among migraine types and preventive treatments. A multivariate linear regression analysis between the percent change in the primary outcome measure and predictive factors was then performed. We also computed the odds ratio for the primary outcome, considering gender, presence of allodynia and comorbidity with fibromyalgia.

## Results

### Demographic data for selected patients

**Fig. 1:**
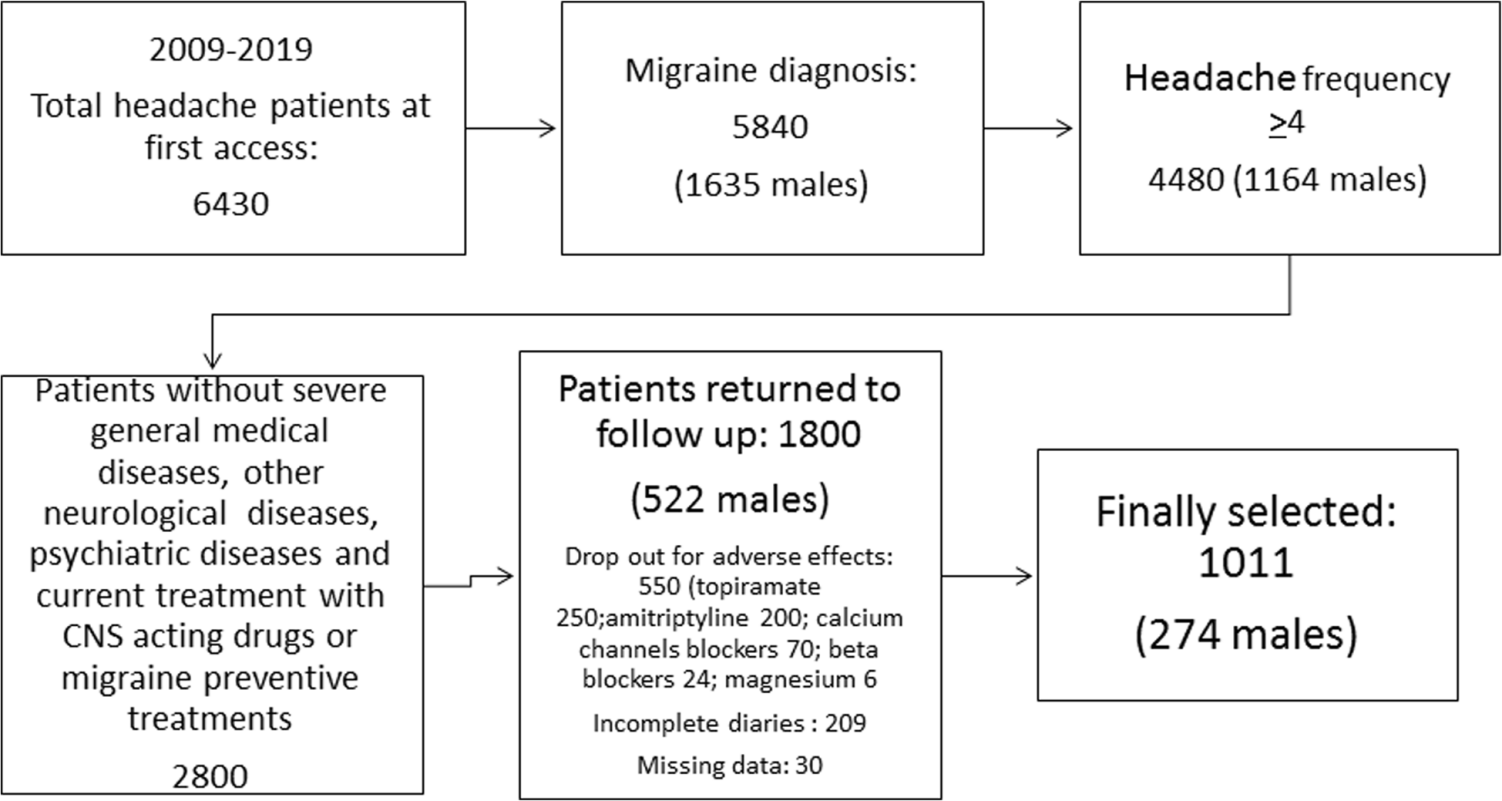
Flowchart depicting migraine patient selection criteria

The flowchart depicting patient selection is shown in Fig. 1. Among a total of 4480 migraine patients with high-medium headache frequency, 2800 met the selection criteria, while 550 were under current or had previously received preventive treatments; 320 were using CNS-targeting drugs for psychiatric comorbidity; 540 reported other primary headaches in association with migraine such as tension-type headache or primary stabbing headache; 102 had other neurological diseases, such as multiple sclerosis, polyneuropathies, previous cerebrovascular disorders, dementia and myasthenia; and the remainder were affected by severe general medical diseases (Fig. 1). Two hundred fifty-five patients used triptans for symptomatic treatment, and the remaining patients used NSAIDS. All patients were given suggestions to take triptans and/or NSAIDs (400–600 mg of ibuprofen) for migraine attacks. Patients with medication overuse were requested to replace the abused drug and to seek symptomatic therapy only in cases of severe headache.

The demographic and main clinical data are reported in Table 1. Patients who dropped out because of adverse events were similar in age and sex to the selected group (38.12 ± 12.1; 135 males). The adverse effects of topiramate were sedation (225 patients), paresthesia (24 patients) and weight loss (1 patient); those for amitriptyline were sedation (150 patients), tachycardia and arrhythmia (15 patients), irritability and insomnia (15 patients) and weight gain (20 patients); those for flunarizine were weight gain (55 patients) and sedation (15 patients); those for beta-blockers were hypotension and bradycardia (24 cases); and those for magnesium were gastrointestinal symptoms (6 patients). No patients requested hospitalization. Among the 296 patients with chronic migraine, 202 reported the use of more than 10 monthly doses of NSAIDs, so they received a diagnosis of medication-overuse headache (MOH) [9]. CM patients presented with older age and higher anxiety and depression scores than the other participants (Table 1).

**Table 1:** Demographic and clinical data of migraine patients

			Mean	SEM	95% CI		Post hoc *(Bonferroni)*
		cases			Lower	Upper	
age ANOVA F: 9.08, *P* < 0.0001	MO	176 M; 471 F	37.49	0.52	36.46	38.51	Vs CM *p* < 0.001
	MA	3 M; 9 F	28.55	3.91	20.86	36.23	Vs CM p 0.005
	MO + MA	17 M; 39 F	35.91	1.89	32.20	39.63	Vs CM p 0.009
	CM	78 M; 218 F	40.94	0.79	39.40	42.49	
migraine duration ANOVA F: 1.44, P 0.22	MO		15.53	0.47	14.62	16.45	n.s.
	MA		11.27	3.49	4.42	18.12	
	MO + MA		16.74	1.69	13.43	20.06	
	CM		16.70	0.70	15.32	18.08	
headache frequency ANOVA F: 238.39, *P* < 0.0001	MO		9.61	0.27	9.08	10.13	Vs CM *p* < 0.001
	MA		6.82	2.00	2.90	10.74	Vs CM *p* < 0.001
	MO + MA		9.24	0.97	7.35	11.14	Vs CM *p* < 0.001
	CM		22.26	0.40	21.47	23.05	
MIDAS ANOVA F: 27.93, *p* < 0.0001	MO		25.62	1.70	22.29	28.95	Vs CM *p* < 0.001
	MA		18.82	12.68	−6.06	43.70	Vs CM p 0.048
	MO + MA		20.77	6.13	8.73	32.80	Vs CM *p* < 0.001
	CM		52.99	2.55	47.99	58.00	
headache intensity (0–100 VAS) ANOVA F: 4.67, p 0.005	MO		8.74	0.05	8.65	8.83	Vs CM p 0.007
	MA		8.82	0.34	8.14	9.49	
	MO + MA		9.09	0.17	8.76	9.41	
	CM		9.04	0.07	8.90	9.17	
Allodynia F: 0.80, p 0.01	MO		2.67	0.08	2.51	2.82	n.s.
	MA		1.82	0.60	0.64	3.00	
	MO + MA		3.15	0.29	2.58	3.72	
	CM		3.01	0.12	2.78	3.25	
SAS F: 8.57, *p* < 0.001	MO		36.68	0.37	35.97	37.40	Vs CM *p* < 0.001.
	MA		35.64	2.75	30.24	41.04	
	MO + MA		36.04	1.28	33.53	38.55	Vs CM p 0.035
	CM		39.88	0.55	38.81	40.95	
SDS F: 11.2, *p* < 0.001	MO		35.28	0.38	34.52	36.03	Vs CM *p* < 0.001.
	MA		30.36	2.90	24.68	36.05	Vs CM: p 0.023
	MO + MA		34.82	1.35	32.18	37.47	Vs CM p 0.03
	CM		38.94	0.57	37.82	40.07	

*MO* migraine without aura, *MA* migraine with aura, *CM* chronic migraine. Frequency: average number of days with headache per month over the 3 months preceding the first visit. *SAS* Zung anxiety score. *SDS* Zung depression score. The results of ANOVAs (DF = 3) and post hoc Bonferroni tests are reported

Table 2 reports the preventive treatments used by all patients. Fifty patients who were treated with antidepressants, 66 with antiepileptics, 2 with beta-blockers, 5 with calcium channel blockers and 1 with sartans reported slight side effects and did not request drug suspension. Eight hundred and twenty patients used triptans with good effects; 220 continued to use NSAIDs, while in the remaining patients, neither drug was efficacious.

**Table 2:** Preventive drugs used by migraine patients

Drugs	Daily Dosages	Migraine diagnosis				Total
		MO	MA	MA + MO	CM	
Beta-blockers	Propranolol: 80–160 mg; atenolol: 50–100 mg	36	0	2	7	45
calcium channel blockers	Flunarizine: 5 mg	139	2	9	15	165
Antidepressants	Amitriptyline: 10–25 mg	245	5	17	158	425
Integrators	Magnesium: 300–400 mg	57	0	4	3	64
Antiepileptics	Topiramate: 50–100	149	5	22	100	276
Sartans	Candesartan: 8–16 mg	21	0	2	13	36
Total		647	12	56	296	1011

The average frequency of migraine at baseline differed among the drugs prescribed: it was higher in the groups treated with antiepileptics and antidepressants than in the other groups, excluding the group prescribed sartans (Table 3). For 154 females presenting with FM comorbidity, neurologists suggested amitriptyline in 104 cases, topiramate in 25 cases, flunarizine in 5 cases, propranolol in 2 cases, magnesium in 2 cases and sartans in 3 cases. Amitriptyline was preferred for patients with mild anxiety and depression (Table 1S).

**Table 3:** Frequency of headache at baseline in the different drug groups. ANOVA with drug as a factor: *F* = 20.54, *p* < 0.0001. Bonferroni with antiepileptics and antidepressants vs beta-blockers, calcium channel blockers and integrators: *p* < 0.001. Sartans vs integrators: *p* < 0.05

Drugs	Mean	SEM	95% CI	
			Lower	Upper
Beta-blockers	9.46	1.25	7.01	11.90
Calcium channel blockers	9.45	0.65	8.17	10.73
Antidepressants	14.64	0.41	13.84	15.43
Integrators	7.30	1.05	5.25	9.36
Antiepileptics	15.24	0.50	14.25	16.23
Sartans	12.94	1.40	10.21	15.68

**Fig. 2:**
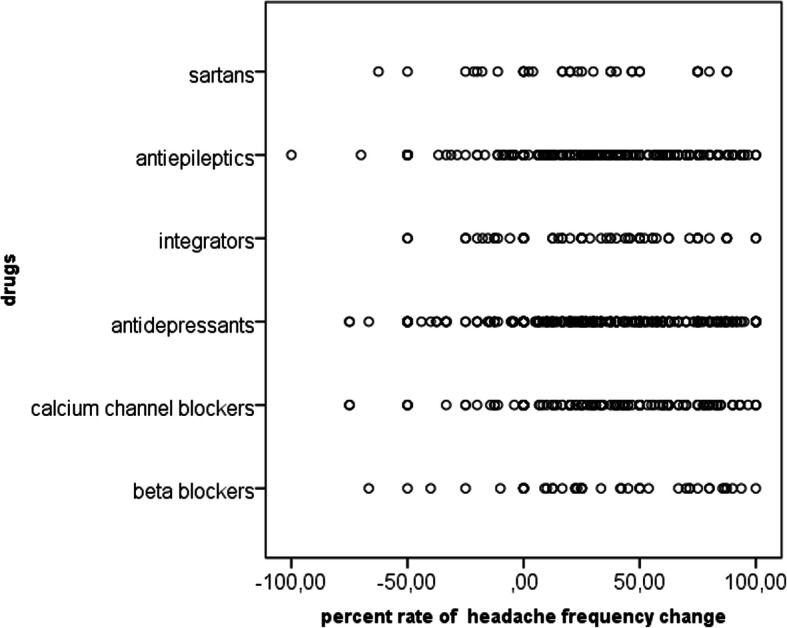
Representation of the percent change in headache frequency for single cases

The mean reductions in headache frequency and confidence intervals are reported in Table 4 and Table 2S. Most of the patients experienced a reduction in headache frequency of less than 50%, with a slight increase in patients with a favorable outcome in the group treated with flunarizine (Table 5; Fig. 2). The exclusion from the analysis of patients with FM comorbidity, which could have influenced the choice of drugs, did not substantially change the percent change in responders (odds ratio: 0.94, p: 0.56), except for the lack of statistical significance for the calcium channel blocker effect (Table 3S). One hundred eighty CM patients with associated MOH discontinued the previous symptomatic drug, instead using the suggested therapy (triptans). One hundred fifty-five CM patients persisted as chronic, the remaining CM patients shifted to a diagnosis of episodic migraine, and in all of these patients, the diagnosis of MOH was not confirmed. The 40 remaining patients continued to use NSAIDs in excess, and the diagnosis of CM with MOH was confirmed.

**Table 4:** Mean (M) and standard deviation (SD) of the percent reduction in migraine frequency for patients with MO: migraine without aura, MA: migraine with aura, and CM: chronic migraine

Drugs	Migraine diagnosis	M (%)	DS	Case number
Beta-blockers	MO	23.31	63.17	36
	MO + MA	12.50	17.68	2
	CM	56.67	40.60	7
	Total	28.02	59.72	45
calcium channel blocker	MO	34.04	40.41	139
	MA	56.25	61.87	2
	MO + MA	44.40	38.54	9
	CM	25.06	29.09	15
	Total	34.05	39.51	165
Antidepressants	MO	26.93	43.49	245
	MA	20.45	36.63	5
	MO + MA	22.09	56.62	17
	CM	31.05	32.88	157
	Total	28.18	40.37	424
Integrators	MO	25.88	54.23	57
	MO + MA	33.33	22.82	4
	CM	27.92	24.25	3
	Total	26.44	51.59	64
Antiepileptics	MO	18.51	56.06	149
	MA	56.59	34.73	5
	MO + MA	22.87	58.33	22
	CM	29.97	32.88	101
	Total	23.70	49.05	276
Sartans	MO	22.35	57.85	21
	MO + MA	25.00	35.36	2
	CM	16.45	23.65	13
	Total	20.37	46.36	36
Total	MO	26.07	48.84	647
	MA	41.47	40.24	12
	MO + MA	26.54	50.79	56
	CM	30.31	32.64	295
	Total	27.52	44.75	1011

ANOVA with drugs as a factor: *F* = 0.47, *p* = 0.81; with migraine diagnosis as a factor: *F* = 0.73, *p* = 0.53; with drugs x migraine diagnosis: *F* = 0.70, *p* = 0.73

**Table 5:** Number of patients with favorable (reduction in headache frequency >  50%) and unfavorable (< 50%) outcomes after 3 months of treatment. The odds ratio was computed for a single drug compared with the remaining population

	Beta-blockers	Calcium channel blockers	Antidepressants	Integrators	Antiepileptics	Sartans	Total
< 50%	28	98	288	40	196	27	677
> 50%	17	67	137	24	80	9	334
	45	165	425	64	276	36	1011
Odds ratio	1.23	1.48	0.93	1.23	0.77	0.66	
95% CI	0.67 to 2.30	1.05 to 2.08	0.71 to 1.2	0.71 to 2.08	0.57 to 1.046	0.3 to 1.4	
z statistic	0.69	2.25	0.461	0.78	1.67	1.037	
Sig. level	*P* = 0.48	*P* = 0.02	*P* = 0.64	*P* = 0.43	*P* = 0.09	*P* = 0.29

The MANOVA comparing secondary outcome variables among different treatments and migraine subtypes showed a general improvement at the 3-month follow-up, which was not different among the preventive treatments and migraine diagnoses. The within-subject analysis showed significant reductions in headache intensity and allodynia in all treated patients (Table 6).

**Table 6:** Mean (M) and standard deviation (SD) of secondary outcome variables in 1011 migraine patients at baseline and after 3 months under preventive treatment (demographic data are reported in Table 1)

Condition		MIDAS	VAS	ALLODYNIA	PH SF36	MH SF36
Baseline	M	33.30	8.82	2.81	36.59	38.67
	SD	43.97	1.16	2.00	8.93	8.75
Follow-up	M	21.74	8.30	2.40	38.10	39.48
	SD	31.22	1.46	1.98	8.74	8.99
Within-subject ANOVA	F	2.93	9.39	3.39	1.14	1.37
	DF	2	2	2	2	2
	P	0.053	< 0.001	0.034	0.31	0.25

MANOVA, condition (baseline vs follow-up): F (Roy square) = 5.12, DF = 5, *p* < 0.0001; condition vs drug: *F* = 0.64, DF = 5, *P* = 0.69; condition vs migraine type: F = 1.83, DF = 5 *p* = 0.1; condition vs drug vs migraine type: *F* = 0.55, DF = 12, *p* = 0.88. The within-subject ANOVA results are reported

*PH* physical Health score SF36, *MH* mental health score SF36

**Fig. 3:**
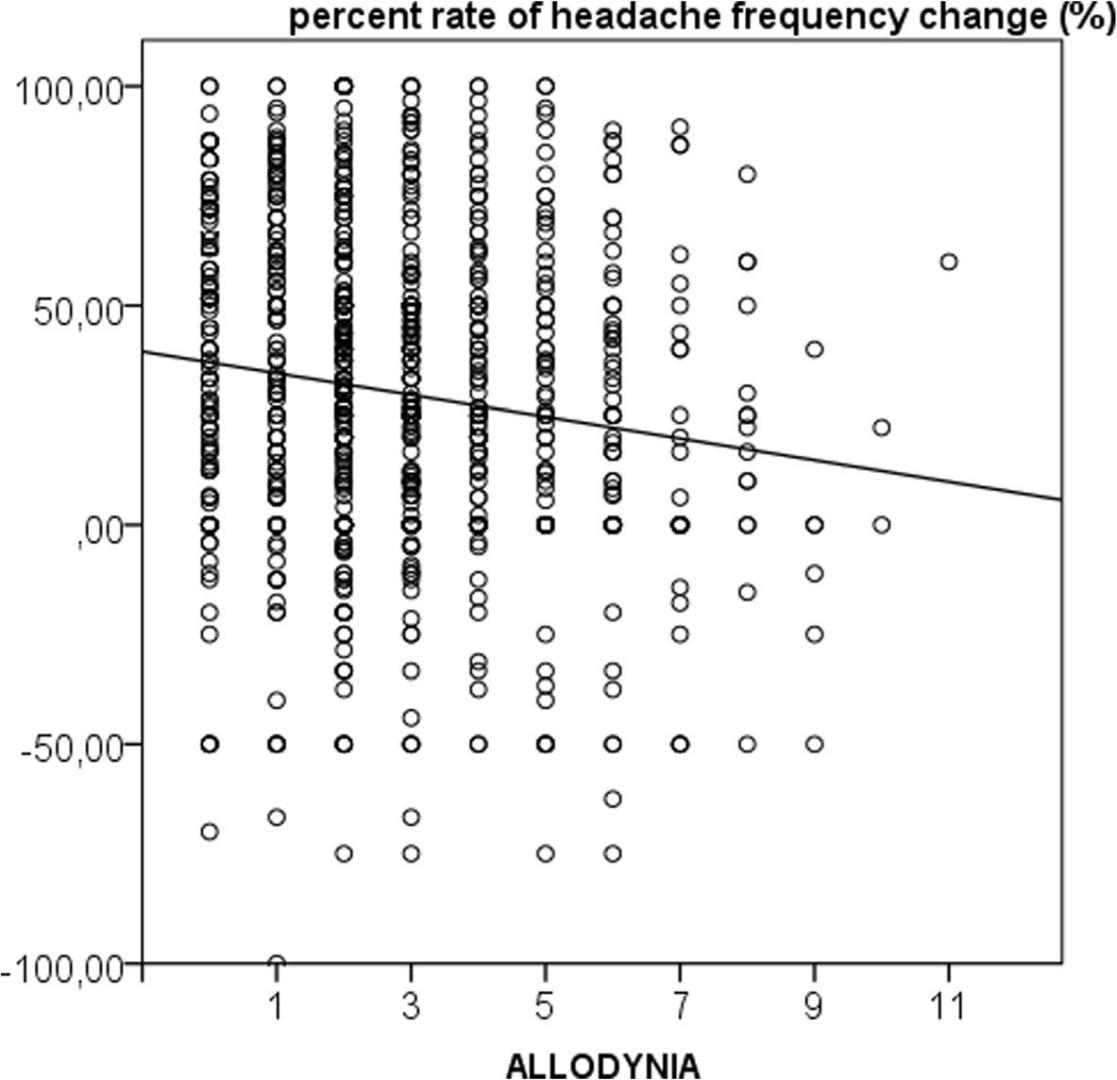
Linear regression analysis between the rate of headache frequency change and allodynia at baseline

The multiple regression analysis showed that a lower allodynia score and a lower frequency of headache at baseline predicted a favorable outcome with at least a 50% frequency reduction (Table 7, Table 4S; Fig. 3). The relationship between allodynia score and headache frequency was confirmed for antiepileptics, while in the group treated with antidepressants, a lower allodynia score and a lower headache intensity were associated with a better outcome (Table 5S). The multiple regression analysis did not show relevant results for the groups treated with the other drugs.

**Table 7:** Multiple regression analysis of the change in frequency at follow-up in 1011 migraine patients

	Nonstandardized coefficients		Standardized coefficients	t	Sig.
	B	Standard deviation (error)	Beta		
(Constant)	49.46	12.72		3.89	0.0001
SAS	−0.37	0.23	−0.08	−1.58	0.11
SDS	0.03	0.23	0.01	0.14	0.89
VAS	−0.47	1.27	−0.01	− 0.37	0.71
Frequency at baseline	0.65	0.17	−0.13	−3.83	0.001
Allodynia	−3.02	0.79	−0.13	−3.81	0.001
AGE	−0.13	0.13	−0.04	−1.02	0.31
Illness duration	0.00	0.15	0.00	0.01	0.99

*SAS* Zung anxiety score

*SDS* Zung depression score

Comorbidity with FM and the presence of allodynia were associated with a lower number of patients with a good outcome, while gender had no effect on drug efficacy (Table 8).

**Table 8:** Effect of fibromyalgia (FM) comorbidity, gender and allodynia on the primary outcome (50% headache frequency reduction)

		Outcome		Odds ratio	95% CI	z statistic	Significance level
		< 50%	> 50%				
FM comorbidity	no	571	299	1.58	1.05 to 2.38	2.2	P = 0.02
	yes	106	35				
Gender	M	180	94	1.08	0.8 to 1.44	0.523	P = 0.6
	F	497	240				
Allodynia	No	73	67	0.5	0.35 to 0.72	3.67	*P* = 0.0002
	Yes	594	277				

## Discussion

This observational retrospective cohort study tested the effects of preventive treatments in a population of migraine patients visiting a tertiary headache center.

The main results consisted of a mild effect of treatments on headache frequency, with a less than 50% reduction in most cases, as well as on migraine-related disability and general quality of life. Flunarizine was prescribed for patients with a lower headache frequency, rather than antiepileptics and antidepressants, and showed slight superiority in terms of therapeutic efficacy. Amitriptyline was preferred for patients with higher anxiety and depression scores.

The absence of or less severe allodynia predicted a better outcome, while comorbidity with fibromyalgia was associated with a reduced therapeutic effect.

### General considerations regarding headache populations

These real-life data show that in our tertiary headache center in South Italy, most of the patients suffered medium-high frequency migraine, they were not previously treated with preventive drugs, and only a minority of them used triptans. An Italian study [3] showed that only a minority of patients using triptans and that qualified for prophylaxis used preventive drugs. Therefore, they did not have access to headache specialists or centers, minimally complied with the prescribed treatment, or withdrew because of adverse events. Our impression is that most of the patients were referred to our center for medium-high frequency migraine that was previously underestimated, though it warranted preventive treatment and triptan prescription. This finding is in line with the results of a study conducted 10 years ago with data from 10 Italian headache centers, which demonstrated that only 26.8% of 2675 patients attending the centers had previously received a diagnosis of migraine [15].

Of the total number of patients free from other CNS drugs or relevant comorbidities, approximately 1000 were lost to follow-up, in accordance with previous studies [16, 17]. Poor adherence is recognized as the major factor impairing the efficacy of migraine prophylactics [18]. More than 500 patients withdrew because of adverse events, which confirms that low compliance may be attributed to side effects and the low efficacy of prescribed drugs.

In our selected migraine sample, only a minority suffered medium-high-frequency migraine with aura, which is in accord with data for the general population [19, 20].

The duration of migraine was very long in our sample, even in several patients without previous preventive treatments. Patients with CM were naïve to treatment, although their clinical picture was obviously more severe than that of the episodic migraine groups, with more severe disability and higher anxiety and depression scores. It is thus conceivable that transformation to chronic migraine recently occurred, although most of these patients had a history of drug abuse, according to current knowledge [21].

The neurologists treated patients in accordance with Italian guidelines [2]. Sodium valproate was reserved as a second-line approach, as its use is off-label in Italy. The choice of antidepressants and antiepileptics as a first-line therapy was reserved for patients with more severe and chronic migraine, according to previous studies [21, 22]. The use of magnesium in 64 patients as a preventive treatment deserves proper discussion. Nutraceutics have low efficacy in migraine prophylaxis, and their level of evidence is very low [23, 24]. Moreover, migraine patients are not confident in the use of CNS-targeting drugs [25], and sometimes they do not agree with this approach. The use of magnesium and other nutraceutics is frequent in clinical practice [23]. This is why we decided to include patients with magnesium treatment in the final analysis. According to current national guidelines [2], candesartan has a recommendation level of 3, but clinicians used it in only a small number of patients. A review of clinical records revealed that patients had mild but underestimated and uncontrolled hypertension and contraindications to the use of beta-blockers. Considering that this situation may occur in clinical practice, we also included this group in the final analysis.

### Effects of preventive treatments on migraine frequency

There was only a mild effect on headache frequency in the 3 months following preventive drug prescription, in accord with the findings of previous studies based on indirect evidence [3, 16, 21]. The failure of the first preventive attempt was also confirmed in 80% of patients in a large population with migraine (9856 patients) interviewed about the burden of the disease [26]. This suggests that there is a difference between randomized controlled trials (RCTs) and real life. Moreover, relevant RCTs are limited, and they were conducted many years ago in small patient series [2]. It is thus not surprising that magnesium was not inferior in efficacy when compared to recommended drugs, as its mild effect confirmed the results of RCTs [23]. In addition, candesartan exerted a slight effect on headache frequency that was not inferior to that of other drugs [27]. The comparison among groups with different case numbers is quite unreliable from a statistical point of view (see paragraph below), but it could support the general impression of weak action of all the prescribed drugs against headache frequency.

Flunarizine at a 5 mg dosage was more efficacious than other drugs in treating headache frequency [28], though the statistical relevance vanished with the removal of patients with FM comorbidity. Moreover, clinicians prescribed flunarizine to patients with a lower frequency of migraine, which is a factor predisposing them to a better outcome. Patients reported the use of triptans in the majority of cases, which could be partly responsible for the reduction in headache intensity. Chronic patients with NSAID overuse were also invited to shift to triptans. In any case, the majority of patients remained chronic with the overuse of triptans due to the weak effect of preventive treatments.

### Effects of preventive treatments on other clinical variables

The general improvement of migraine-related disability, quality of life, headache intensity and allodynia was independent of the drug and type of migraine. Considering the single variables, the intensity of headache and allodynia changed in a relevant way, an effect attributable to the preventive treatments and probably to the use of triptans. An effect of antiepileptics and antidepressants on allodynia was also reported in previous studies [29]. An Italian longitudinal study on migraine evolution over 3 months of observation reported an evident trend toward improvement in disability and social activity, independent of the use of preventive treatments [30]. The slight improvement of the evaluated clinical features can be attributed partly to spontaneous evolution in patients during continued care and partly to an effect of preventive and acute treatments.

### Predictor variables

Low frequency of migraine and reduced expression or absence of allodynia symptoms at baseline predicted a better effect of preventive treatments in all patients. Studies focusing on factors predicting the effects of acute treatments show that allodynic patients have a worse response to triptans [31]. A predictive role of allodynia was observed in the groups of patients treated with antidepressants and antiepileptics, which included a reliable number of patients. Anxiety and depression at baseline, which were more highly expressed in the CM group, were not associated with a worse efficacy of treatments against headache frequency. We excluded cases with previously documented psychiatric diseases. Considering that scores > 50 for the depression scale and > 45 for the anxiety scale [12, 13] indicate relevant symptoms, our patients presented with scores within normal ranges; however, the scores were higher in our CM patients than in the other participants. Basal anxiety and depression scores do not seem relevant for the final effects of drugs and certainly are not relevant in patients without psychiatric disorders.

Gender was also unrelated to the effect of preventive drugs. Males accounted for 27% of the total selected population, which is consistent with migraine representation in a sample of the Italian population [32]. Previous studies reported that males have better compliance with treatments than females, though the effect of sex on treatment efficacy was not evaluated [33].

Comorbidity with FM was also associated with a reduced effect of preventive drugs on headache frequency. Most migraine patients with associated FM used amitriptyline, which may be a good therapeutic option when this comorbidity is present [14]. In previous studies, we observed an association between FM and more severe migraine [14]. The present results also confirmed a minor response to preventive treatments. FM patients generally have a weak response to different treatments, indicating the need for an individualized therapeutic approach. In migraine patients with FM comorbidity, central sensitization is a predominant phenomenon, causing a diffusion of pain in somatic sites [14]. In light of the present study, we suggest that the clinical expression of central sensitization phenomena such as allodynia and FM comorbidity predicts a worse response to preventive treatment.

### Study limitations

This observational retrospective study has several limitations.

The sample size for the first outcome variable was reliable for the entire group and for patients treated with antidepressants, while the case number was smaller for the other drugs. Moreover, in our opinion, the results could shed light on real-life situations.

Potential confusion and bias may have affected the final reliability of the data. We found the lack of information about the efficacy of symptomatic treatments particularly important, meaning that the reduction in allodynia and intensity of headache at follow-up cannot be assigned to triptans or preventive treatments.

We focused on fibromyalgia comorbidity, as our center has specific experience in the application of diagnostic criteria. Other comorbidities, such as hypertension or obesity, lifestyle, physical inactivity, habits, smoking, or even professions, could affect the outcome of treatments, but we decided to focus on the main clinical and demographic aspects in selected patients during their first preventive approach, reserving a global evaluation of these factors for further analyses. In addition, the consideration of FM comorbidity may have influenced the choice of drugs, though the removal of such patients did not substantially change the percentage of responders.

The study is observational and lacks a control population, which would be useful for dissecting the effect of drugs from spontaneous evolution.

## Conclusions

A mild effect of current preventive treatments on headache frequency and disability emerged from the present data. The number of patients lost to follow-up or who dropped out because of adverse events was large and confirms that the preventive approach to migraine treatment is currently inefficacious and unwelcome, even in tertiary headache centers. The low number of chronic patients reverting to the episodic form by the three-month follow-up confirms this impression. No drug demonstrated high efficacy, except for flunarizine, which was prescribed to less affected patients. Potential useful indicators for the use of current first-line preventive drugs may be a low frequency of migraine and the absence of allodynia and other central sensitization symptoms, such as fibromyalgia. These results support the early use of more recent treatments such as botulinum toxin or CGRP antagonists in naïve patients with medium-severe headache frequency and allodynia, in contrast to the general trend of offering these as first-line preventive drugs for resistant patients.

## Supplementary information

**Additional file 1.**

**Additional file 2: Table S1.** Zung Anxiety and Depression Scores at baseline in migraine patients submitted to different preventive treatments. Results of MANOVA analysis F 5.49 *p* < 0.0001. Post hoc Bonferroni test: antidepressant vs Calcium Channel blockers and integrators: *p* < 0.01. **Table S2.** 95% Confidence Intervals values for change of headache frequency. **Table S3.** Number of patients without FM comorbidity with favorable (reduction of headache frequency >  50%) and unfavorable (< 50%) outcome after 3 months. The odd ratio was computed for single drugs, compared with the remaining population. **Table S4.** Details of multiple regression analysis for change of headache frequency. **Table S5.** Multiple regression analysis between clinical features at baseline and change of headache frequency in subgroups of migraine patients sas: Zung anxiety score; sds: Zung depression score.

## Data Availability

The datasets used and/or analyzed during the current study are available from the corresponding author upon reasonable request.

## Abbreviations

MA: Migraine with aura
MO: Migraine without aura
CM: Chronic migraine
FM: Fibromyalgia
SDS: Self-rating depression scale
SAS: Self-rating anxiety scale
