# Supplementary figures and images for: Failure of preventive treatments in migraine: an observational retrospective study in a tertiary headache center
Source: BMC Neurol. 2020 Jun 30;20:256. doi: 10.1186/s12883-020-01839-5 (PMC7345518; doi:10.1186/s12883-020-01839-5)

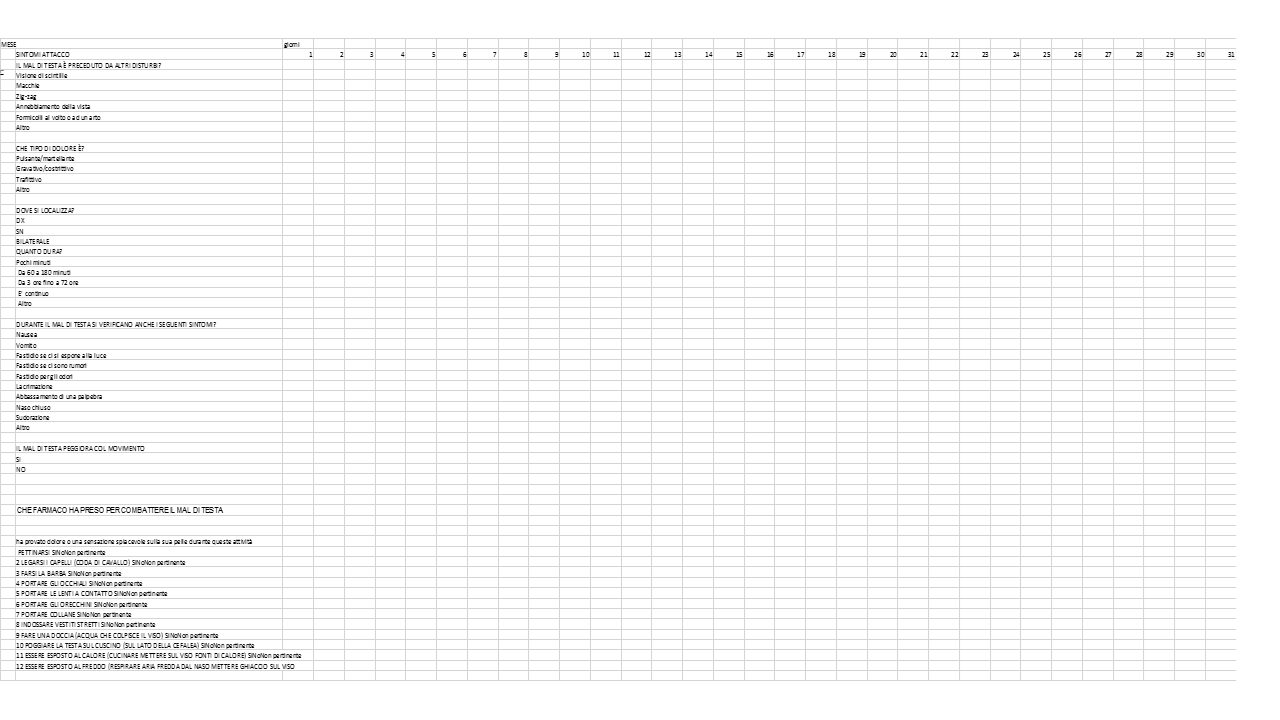

Supplement: Supplementary file 1 — Additional file 1. [file 12883_2020_1839_MOESM1_ESM.tif]
