# Supplementary material for: Failure of preventive treatments in migraine: an observational retrospective study in a tertiary headache center
Source: BMC Neurol. 2020 Jun 30;20:256. doi: 10.1186/s12883-020-01839-5 (PMC7345518; doi:10.1186/s12883-020-01839-5)
Supplement: Supplementary file 2 — Additional file 2: Table S1. Zung Anxiety and Depression Scores at baseline in migraine patients submitted to different preventive treatments. Results of MANOVA analysis F 5.49 p < 0.0001. Post hoc Bonferroni test: antidepressant vs Calcium Channel blockers and integrators: p < 0.01. Table S2. 95% Confidence Intervals values for change of headache frequency. Table S3. Number of patients without FM comorbidity with favorable (reduction of headache frequency > 50%) and unfavorable (< 50%) outcome after 3 months. The odd ratio was computed for single drugs, compared with the remaining population. Table S4. Details of multiple regression analysis for change of headache frequency. Table S5. Multiple regression analysis between clinical features at baseline and change of headache frequency in subgroups of migraine patients sas: Zung anxiety score; sds: Zung depression score. [file 12883_2020_1839_MOESM2_ESM.docx]

|  | Drugs | Mean | Error DS | 95% CI | |
| --- | --- | --- | --- | --- | --- |
|  |  |  |  | Lower | Upper |
| SAS | Beta Blockers | 36.333 | 1.389 | 33.608 | 39.059 |
|  | Calcium Channel Blockers | 33.601 | .716 | 32.196 | 35.007 |
|  | Anidepressants | 39.553 | .446 | 38.677 | 40.428 |
|  | Integrators | 35.169 | 1.172 | 32.870 | 37.469 |
|  | Antiepileptics | 37.618 | .551 | 36.537 | 38.699 |
|  | Sartans | 37.412 | 1.544 | 34.382 | 40.441 |
| SDS | Beta Blockers | 35.167 | 1.477 | 32.267 | 38.066 |
|  | Calcium Channel Blockers | 32.734 | .762 | 31.239 | 34.229 |
|  | Anidepressants | 38.022 | .475 | 37.091 | 38.953 |
|  | Integrators | 32.814 | 1.246 | 30.367 | 35.260 |
|  | Antiepileptics | 36.487 | .586 | 35.337 | 37.637 |
|  | Sartans | 37.059 | 1.642 | 33.837 | 40.281 |

Table 1 S: Zung Anxiety and Depression Scores at baseline in migraine patients submitted to different preventive treatments

Results of MANOVA analysis F 5.49 p <0.0001

Post hoc Bonferroni test: antidepressant vs Calcium Channel blockers and integrators: p<0.01.

| Change of frequency of headache (%) | | 95% CONFIDENCE INTERVALS | |
| --- | --- | --- | --- |
| Preventive Treatments | Diagnosis | Lower Limit | Upper Limit |
| Beta blockers | MO | 8,672 | 37,941 |
|  | MA |  |  |
|  | MO+MA | -49,591 | 74,591 |
|  | CM | 23,481 | 89,858 |
| Calcium Channel Blockers | MO | 26,589 | 41,485 |
|  | MA | -5,841 | 118,341 |
|  | MO+MA | 15,128 | 73,668 |
|  | CM | 2,383 | 47,728 |
| Antidepressants | MO | 21,315 | 32,535 |
|  | MA | -18,822 | 59,717 |
|  | MO+MA | ,791 | 43,384 |
|  | CM | 24,042 | 38,058 |
| Integrators | MO | 14,248 | 37,510 |
|  | MA |  |  |
|  | MO+MA | -10,571 | 77,238 |
|  | CM | -22,780 | 78,613 |
| Antiepileptics | MO | 11,317 | 25,704 |
|  | MA | 17,320 | 95,859 |
|  | MO+MA | 4,144 | 41,586 |
|  | CM | 21,194 | 38,756 |
| Sartans | MO | 3,193 | 41,516 |
|  | MA |  |  |
|  | MO+MA | -37,091 | 87,091 |
|  | CM | -7,901 | 40,807 |

Table 2S: 95% Confidence Intervals values for change of headache frequency.

Table 3 S Number of patients without FM comorbidity with favorable (reduction of headache frequency >50% ) and unfavorable (<50%) outcome after 3 months. The odd ratio was computed for single drugs, compared with the remaining population.

|  | beta blockers | calcium channel blockers | antidepressants | Integrators | antiepileptics | Sartans |  |
| --- | --- | --- | --- | --- | --- | --- | --- |
| <50% | 26 | 95 | 215 | 38 | 175 | 24 | 573 |
| >50% | 16 | 65 | 111 | 24 | 74 | 9 | 299 |
|  | 42 | 160 | 326 | 62 | 219 | 36 | 1011 |
| Odds ratio | 1.17 | 1.31 | 0.98 | 1.21 | 0.81 | 0.71 |  |
| 95 % CI: | 0.62 to 2.23 | 0.92 to 1.85 | 0.75 to 1.29 | 0.71 to 2.05 | 0.59 to 1.01 | 0.3 to 1.5 |  |
| z statistic | 0.5 | 1.53 | 0.078 | 0.7 | 1.34 | 0.83 |  |
| Sig. level | P = 0.61 | P =0.12 | P = 0.93 | P = 0.48 | P = 0.17 | P = 0.47 |  |

|  | R | corrected R-square | Standard error |  |  |
| --- | --- | --- | --- | --- | --- |
|  | ,178^a^ | ,024 | 35,01967 |  |  |
| **Anova^a^** | | | | | |
| Model | | df | Sum of squares | F | Sig. |
| 1 | Regression | 7 | 5359,631 | 4,370 | ,000^b^ |
|  | Residual | 1003 | 1226,377 |  |  |
|  | Total | 1011 |  |  |  |
| a.dependenta variable: percent rate of headache frequency change | | | | | |
| b. Predictors (Constant),migraine duration, frequency at baseline, VAS, Allodynia at baseline, age, anxiety and depression scales | | | | | |

Table 4S: Details of multiple regression analysis for change of headache frequency .

| **Coefficients** | | | | | | | | |
| --- | --- | --- | --- | --- | --- | --- | --- | --- |
|  | | Not standardized | | standardized | t | Sig. | 95.0% CI for B | |
|  |  | B | Error DS | Beta |  |  | Lower | Upper |
| Antidepressants  ( 425 cases) | Costant | 57.570 | 15.537 |  | 3.705 | .000 | 27.024 | 88.116 |
|  | Age | .160 | .153 | .062 | 1.046 | .296 | -.141 | .461 |
|  | Illness duration | -.042 | .177 | -.014 | -.240 | .810 | -.390 | .305 |
|  | Sas | -.113 | .277 | -.029 | -.408 | .683 | -.658 | .432 |
|  | Sds | .007 | .267 | .002 | .027 | .978 | -.517 | .532 |
|  | frequency at baseline | .306 | .194 | .080 | -1.580 | .115 | -.075 | .686 |
|  | Allodynia | -2.459 | 1.022 | -.131 | -2.407 | .017 | -4.468 | -.450 |
|  | Vas | -3.098 | 1.513 | -.103 | -2.048 | .041 | -6.071 | -.124 |
| Antiepilepileptics  (276 cases) | Costant | 27.239 | 19.811 |  | 1.375 | .170 | -11.776 | 66.254 |
|  | Age | -.329 | .207 | -.125 | -1.591 | .113 | -.736 | .078 |
|  | Illness duration | .204 | .213 | .072 | .958 | .339 | -.216 | .624 |
|  | Sas | -.165 | .343 | -.045 | -.479 | .632 | -.841 | .512 |
|  | Sds | .092 | .325 | .027 | .282 | .778 | -.548 | .732 |
|  | frequency at baselinie | .605 | .245 | .156 | -2.470 | .014 | -.123 | 1.088 |
|  | Allodynia | -2.409 | 1.082 | -.145 | -2.226 | .027 | -4.541 | -.278 |
|  | Vas | 1.165 | 1.953 | .037 | .597 | .551 | -2.681 | 5.012 |

Table 5S Multiple regression analysis between clinical features at baseline and change of headache frequency in subgroups of migraine patients

sas: Zung anxiety score; sds: Zung depression score
